# Modern Maladies and Present State of Medicine

**Published:** 1818-06

**Authors:** 


### PART II.

### COMPREHENSIVE ANALYTICAL REVIEW OF MEDICAL LITERATURE.

"Tros, tyriusve, nobis nullo discrimine agetur."

*Modern Maladies and present State of Medicine.* By D. Uwins, M. D. Octavo, pp. 43. 1818.

This was the Anniversary Oration delivered March 9th, 1818, before the Medical Society of London, by our respected contemporary Editor, Dr. Uwins.

Our author conceives that the introduction of our vernacular language as the vehicle of medical disqusitions, has impaired the public confidence in medicine, since the clashings of accredited authorities "respecting subjects which actually involve the issues of life and death," are daily blazoned forth in newspapers and magazines. There is no doubt but that among those who are *well* these discussions excite merriment, if not contempt; but let sickness overtake one of the same individuals, and we shall soon see him send as quickly for his physician or other medical attendant as in the darkest ages of ignorance. The impudent and mendacious assertions of Charlatanism, it is true, entrap a certain portion of credulous patients who, in their turn, come back on the regular practitioner with an exasperation of their complaints; still the vernacular writings of well-educated physicians and surgeons have not only diffused scientific information more universally through the various ramifications of the profession, than it otherwise could have circulated in a Greek or Latin dress; but they have enabled the superior orders of society to discriminate genius from dullness—learning from ignorance—talent from mediocrity—and industry from idleness.

Dr. Uwins has, on more than one occasion, ably advocated the cause of "Modern Medicine." For our own parts, we have long been sick of Celsus, as a paragon of knowledge, believing, as we do, that the human *intellect* is just as strong now as in the Augustan age; and human *judgment* infinitely stronger, from the accumulated experience of nearly twenty centuries. But in literature as in landscape—

"'Tis *distance* lends enchantment to the view;"

And we are lost in "astonishment," that *two thousand years ago,* fever should have produced a quick pulse and hot skin; and still more so, that Celsus should have not only recorded such phænomena, but actually prescribed blood-letting and the cold affusion! We could heartily wish to see some one of those who find every modern discovery or improvement in Celsus, at the bed-side of a patient—say in fever, applying the ancient rules of treatment, as they are handed down to us by this eloquent writer. He would find a vast contrast between Celsus and Currie in respect to the cold affusion! He would point out indeed, with pride, how Celsus directs that the patient labouring under *ardent* fever is to be refrigerated with oil and water.—"Sed in ipsis accessionibus oleo et aqua refrigerandus est." *Lib. iii. viii.* 2.—But he would not say a word respecting this same author's precept in *slow continued fevers* [lentæ febres sine ulla remissione] where, in order to produce a *change* in the disease, [ut morbum mutet] cold affusions are to be used, so that a *rigor* may be induced, and a new train of movements instituted! "Sæpe igitur ex aqua frigida, cui oleum sit adjectum, corpus ejus pertractandum est, *quoniam interdum sic evenit, ut horror oriatur, et fiat initium quoddam novi motus.*" *Lib. iii. ix.*—Now we fearlessly assert, that the foregoing precept is not only extremely dangerous and pernicious in itself, but affords a convincing proof that Celsus was totally ignorant of those rules which are to guide us in the application of cold water to the fevered surface; and consequently, as may be seen by the above quotation, that he was more likely to do harm than good with the remedy.

As for *purgatives* in fever, this fountain of all modern improvements nearly proscribes them, and that on the Brunonian plea of *debility!* "Ego autem, medicamentorum dari potiones, *et alvum duci non nisi raro,* debere concedo:—quoniam *imbecillitate* summum periculum est." Lib. iii.—Now, are we patiently to listen to those who would compare these crude—nay, these pernicious precepts of antiquity, with the scientific investigations and deductions of a Currie, a Hamilton, and an Armstrong? Let us but contrast the boldness of a Cooper or Abernethy with the timidity of a Celsus, who shrinks in terror from all wounds.—"In alis vel feminibus, vel *inanibus locis*, vel in articulis, vel *inter digitos:* item quodcunque *musculum, aut nervum, aut arteriam, aut membranam, aut os, aut cartilaginem læsit!*"

We respect the ancients; but not those *laudatores temporis acti*, who are blind to the progress of science; and who, by misconstruing or distorting some ambiguous scraps of antiquity, are perpetually detracting from the merit of modern improvements.

But to return from this digression. Dr. Uwins has drawn a somewhat ludicrous, and we hope and believe a somewhat overcharged portrait of the three "prevailing theories of the present period," viz. the "hepatic" theory, the "digestive organ" theory, and the "vascular irritation" theory. We give the following passage in proof of our opinion that the portraits verge towards the caricature on the first theory.

"Turn up the great lobe of the liver, say the champions of the sect now adverted to, and you will find diseases lying as thick as ants in a mole-hill, which has been disturbed by the scythe of the mower. This is a real Pandora's box, the *origo et causa morborum omnium*; the something which, if you can regulate, you can control disease; if not, disease will bid defiance to all your remedial endeavours. Do you see a child dying with hydrocephalus? What can possibly have produced the derangement, and the approaching death, but something wrong in the liver? Is the disorder tic douloureux, or head-ach, or apoplexy, or epilepsy, or madness, or blindness, see to it that the liver is in a proper state before you either think of cause, or dream of cure. To what other sources than obstructions in the liver can we attribute those affections which have been referred, but erroneously, to primary disorder in the chest? Does the blood find a difficult transmission through the lungs? such difficulty must have been first experienced by the liver. Are there tubercles, or ulcerations, or asthmatic conditions, observable in these organs? How can such tubercle, or ulcer, or asthmatic affection, have originated without the liver having planted their seeds, and regulated their growth? Do we find inflammatory conditions, aneurismal dilatations, organic obstructions in the heart and its great blood-vessels? Who shall pretend that ossification, that obstruction, that dilatation, can have place, unless through the agency of the liver? Stomach and bowel derangements, would our theorists say, are still more obviously and unequivocally our own. Concede this to us, and at the same time observe how intimately connected such ventricular states are with the origin and decline of many other morbid affections; and the inference must be, that all these maladies are, in effect, hepatic. Rheumatic inflammation, for instance, may or may not be an inflammation seated in membranous fascia: but whether it be or be not, it is the liver which has transmitted the blood, charged with powers to create the local disturbance. Again, an individual is attacked with what you please to name gout. How frequent it is to observe such an attack alternating with states in which the liver is undeniably affected in its functions; ergo, do our hepatic logicians infer, gout is resident in the liver. Multiform and various, to be sure, are the disordered irritations to which the kidnies and connected parts are obnoxious. The urine, instead of being poured out from its gland of secretion, with all its healthy products and principles, is sometimes found loaded with a vast proportion of saccharine matter; but it is needless to amuse yourself with fine spun theories of the *quo-modo* of such phenomenon; it is further loss of time, to aim at ascertaining the different qualities and ingredients of calculary formation, or try to find out the *modus operandi* of lithic concretion; it is all—all done by the liver; and looking at any thing else than the liver, we merely investigate incidental effects, instead of being more sensibly and more profitably engaged in raising our contemplation to the source of every thing." P. 17, 18.

The above will serve as a specimen of our author's powers in *taking off* the extravaganzas of modern medical theories. But Dr. Uwins is a good-humoured, and moreover a sensible satirist. He only ridicules the *excess*, not the moderate views of these different theorists. In the following passage, we most cordially and implicitly agree with our well informed author.

"The animal machine, as it appears to me, refuses to be regulated by that simplicity of movement which our desires dictate, and our ingenuity devises: and I would sum up the whole of the evidence, by expressing it as my humble opinion, that there is *nothing faulty* in any of the tenets upon which I have ventured to comment, excepting in their unwarrantable extension and exclusive application. Moderately conceived and discriminately applied, they are all true, and all useful." P. 39.

These are own sentiments. We have been too long acquainted with the complicated structure, functions, and morbid movements of the human frame, together with the complicated and ever-varying etiology of diseases, to think of solving all phænomena on a single principle. But we firmly believe, that the three modifications of doctrine commented on by our author, explain, as far as can yet be explained, the leading phænomena of diseases. To Dr. Curry, Mr. Abernethy, and Dr. Clutterbuck, then, we sincerely believe that the profession owes as much as to any three medical characters that have preceded them in the annals of the healing art.

—— Nihil est ab omni

Parte beatum.——

